# FT3 Levels and Systemic Inflammation: Evidence From a Population‐Based NHANES Analysis

**DOI:** 10.1155/mi/3764432

**Published:** 2026-01-26

**Authors:** Liu Yang, Han-yu Wang, Meng-fei Fu, Yu-han Zhang, Xiao Chen, Zi-xuan Wang, Hui Sun

**Affiliations:** ^1^ Department of Clinical Nutrition, Union Hospital, Tongji Medical College, Huazhong University of Science and Technology, Wuhan, China, hust.edu.cn; ^2^ Department of Endocrinology, Union Hospital, Tongji Medical College, Huazhong University of Science and Technology, Wuhan, China, hust.edu.cn; ^3^ Department of Endocrinology, The Affiliated Hospital of Xuzhou Medical University, Xuzhou, China, xzmc.edu.cn

**Keywords:** FT3, inflammatory markers, NHANES, population-based cross-sectional study, thyroid function

## Abstract

**Objective:**

Previous studies suggest a complex interaction between 3,3′,5‐triiodo‐L‐thyronine (T3) and inflammation, but this relationship remains unclear. This study investigates the association between free triiodothyronine (FT3) levels and inflammatory markers in the US population using National Health and Nutrition Examination Survey (NHANES) data.

**Methods:**

This study analyzed NHANES data from three cycles between 2007 and 2012, using Spearman correlation tests and multivariate linear regression. Subgroup analyses and interaction tests were conducted based on age, gender, race, body mass index (BMI), thyroid‐stimulating hormone, and free thyroxine (T4) to determine the correlations between FT3 and seven systemic inflammatory markers: C‐reactive protein (CRP), neutrophil‐to‐lymphocyte ratio (NLR), platelet‐to‐white blood cell ratio (PWR), platelet‐to‐lymphocyte ratio (PLR), monocyte‐to‐lymphocyte ratio (MLR), systemic immune‐inflammation index (SII), and systemic inflammation response index (SIRI).

**Results:**

A total of 2306 participants were included in this study. Univariate correlation analysis showed that CRP, NLR, MLR, and PLR were significantly negatively correlated with FT3 levels (all *p*  < 0.05). After adjusting for confounders, FT3 levels were significantly negatively associated with CRP, NLR, and PLR (all *p*  < 0.05). Subgroup analysis showed that age significantly modified the associations between FT3 and systemic inflammatory markers, such as CRP, NLR, MLR, SII, and SIRI (*p*‐interaction < 0.05). The inverse associations were more consistent among individuals aged ≥ 65 years.

**Conclusion:**

In the general population, FT3 levels exhibit inverse associations with systemic inflammatory markers.

## 1. Introduction

Thyroid hormones (THs), including 3,3′,5‐triiodo‐L‐thyronine (T3) and thyroxine (T4), are crucial endocrine hormones that regulate human growth, metabolism, and development [1, 2]. Among these, free triiodothyronine (FT3) represents the biologically active form of TH, capable of directly entering target cells and binding to nuclear TH receptors to regulate gene expression and cellular function, thereby exerting its physiological effects [3, 4]. Free T4 (FT4) is primarily a prohormone requiring conversion to active T3 in peripheral tissues, while TSH reflects feedback regulation rather than direct TH action [5]. Therefore, serum FT3 levels more accurately reflect the effective, tissue‐level TH activity compared to either TSH or FT4. Accumulating evidence has demonstrated that alterations in FT3 levels are closely associated with a range of systemic disorders, including cardiovascular diseases, dyslipidemia, type 2 diabetes, and hepatic dysfunction [6–9], highlighting the clinical importance of monitoring FT3 as a functional indicator of thyroid status.

Inflammation serves as a critical defense mechanism against tissue injury or infection; however, chronic inflammation may lead to persistent damage in multiple tissues and organs. Accumulating evidence indicates that systemic chronic inflammation contributes to the pathogenesis of various diseases, including hypothyroidism, diabetes mellitus, metabolic syndrome, cardiovascular diseases, and certain malignancies [10–14]. Chronic inflammation can be reflected by nonspecific systemic inflammatory markers, such as C‐reactive protein (CRP), neutrophil‐to‐lymphocyte ratio (NLR), platelet‐to‐white blood cell ratio (PWR), platelet‐to‐lymphocyte ratio (PLR), monocyte‐to‐lymphocyte ratio (MLR), systemic immune‐inflammation index (SII), and systemic inflammation response index (SIRI). In recent years, systemic inflammatory markers have become an important tool for assessing chronic inflammatory status due to their convenience and low cost, demonstrating significant value in prognostic evaluation across various diseases, including cardiovascular, neurological, infectious diseases, and cancers [15–18].

As research advances, the prognostic value of systemic inflammatory markers in endocrine and metabolic disorders has gained increasing attention. In patients with diabetes, NLR, MLR, and SII have been established as independent predictors of both microvascular and macrovascular complications and are also significantly associated with all‐cause and cardiovascular mortality [19]. In neuroendocrine tumors such as pheochromocytoma, increased levels of inflammatory markers are positively associated with plasma catecholamine metabolites, suggesting a potential role of endocrine hormones in modulating systemic inflammation and supporting the existence of endocrine‐immune crosstalk [20]. Our previous clinical observational study further demonstrated that the lymphocyte‐to‐monocyte ratio (LMR) effectively predicts prognosis in patients with pancreatic neuroendocrine tumors [21]. Collectively, these findings indicate that readily accessible and cost‐effective systemic inflammatory markers are emerging as key biomarkers linking endocrine dysfunction, immune activation, and end‐organ damage, holding significant promise for clinical application.

Emerging evidence has revealed a complex bidirectional relationship between THs and inflammation. Clinical observations demonstrate that low FT3 levels are associated with increased cardiovascular risk and elevated inflammatory markers [22]. In patients with type 2 diabetes mellitus, chronic liver disease, or infectious diseases, decreased FT3 levels frequently coincide with heightened inflammatory status and poor prognosis [23–26]. Animal studies further confirm that T3 can attenuate inflammatory responses in endotoxemia by suppressing NF‐κB signaling pathways [27]. Conversely, proinflammatory cytokines such as Interleukin‐6 (IL‐6) and IL‐1β have been shown to increase the incidence of subacute thyroiditis, Graves’ disease, and Hashimoto’s thyroiditis, subsequently affecting TH levels [28]. Together, these findings underscore the importance of elucidating the clinical interplay between thyroid function and systemic inflammation.

However, current understanding remains limited. Most investigations have focused on specific patient populations (e.g., those with diabetes or liver disease), potentially limiting the generalizability of findings to the general population [25, 26]. Additionally, reliance on single inflammatory markers may fail to reflect the complexity of systemic inflammation. To address these gaps, we analyzed data from the nationally representative National Health and Nutrition Examination Survey (NHANES) to examine the association between FT3 and multiple systemic inflammatory markers in US adults. To our knowledge, this is the first study to integrate a wide spectrum of systemic inflammatory markers to evaluate overall inflammatory status in relation to thyroid function.

## 2. Materials and Methods

### 2.1. Study Population

This study analyzed data from the NHANES, which is a national, population‐based cross‐sectional survey conducted by the National Center for Health Statistics (NCHS). NHANES aims to assess the health and nutritional status of adults and children in the United States, with data collected and released every 2 years. For this study, we retrieved data from NHANES cycles during which TH examinations were performed (2007–2012). The NHANES public‐use datasets were obtained with informed consent signed at the time of data collection and were approved for public use by the NCHS Research Ethics Review Board. The study was conducted in accordance with the Declaration of Helsinki.

### 2.2. Inclusion and Exclusion Criteria

**Figure 1:**
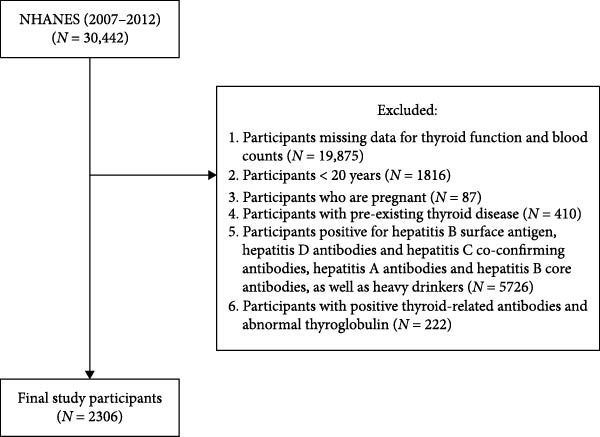
Detailed flowchart for recruiting participants.

Initially, 30,442 participants were identified from the NHANES database. Participants were excluded if they were under 20 years old, pregnant, had a history of thyroid disease, lacked TH or complete blood count (CBC) data, or had positive thyroid‐related antibodies or hepatitis markers. After applying these exclusion criteria, a final sample of 2306 participants was included in the analysis. The detailed selection process is illustrated in Figure 1.

### 2.3. Data Collection

The variables included in this study comprised demographic characteristics, lifestyle factors, and clinical measurements. Specifically, these variables included gender, age, race, alcohol consumption, smoking behavior, body mass index (BMI), systolic blood pressure (SBP) (mmHg), diastolic blood pressure (DBP) (mmHg), FT3 (pg/mL), FT4 (pmol/L), TSH (μIU/mL), alanine aminotransferase (ALT) (U/L), aspartate aminotransferase (AST) (U/L), cholesterol (mmol/L), triglycerides (mmol/L), high‐density lipoprotein (HDL) (mmol/L), fasting blood glucose (mmol/L), uric acid (μmol/L), creatinine (μmol/L), blood urea nitrogen (BUN) (mmol/L), CBC, CRP (mg/dL), and medical history (diabetes, heart disease, hypertension, stroke, and cancer history). Demographic information, including gender, age, and race, was derived from the NHANES demographic dataset. It should be noted that although “RIAGENDR” in NHANES is labeled as “gender,” it is defined based on biological characteristics and reflects sex assigned at birth (male/female). In accordance with NIH guidelines, this variable was treated as biological sex in the present study, and the term “sex” was consistently used throughout the analysis. Race categories included Mexican American, non‐Hispanic White, non‐Hispanic Black, and others. Laboratory data, including TH levels and CBC, were obtained from NHANES laboratory datasets. NHANES has detailed records of laboratory procedures. Blood samples were collected via venipuncture and immediately centrifuged. Specimens were then frozen and transported weekly to central laboratories. Blood samples were processed, stored, and shipped to the Collaborative Laboratory Services in Ottumwa, Iowa. FT3 levels were measured using competitive binding immunoassay methods. CBCs were analyzed using an automated hematology analyzer (Coulter DxH 800 Analyzer). Detailed laboratory methods can be found on the NHANES website.

Personal and medical history data were obtained from questionnaires. Alcohol consumption was defined as having consumed alcohol more than 12 times in the past year, while nondrinkers had fewer than 12 drinks in the past year. Heavy drinking was defined as more than one drink per day for women and more than two drinks per day for men. Smoking status was categorized as smokers if participants reported smoking more than 100 cigarettes in their lifetime; otherwise, they were classified as nonsmokers. Medical history was determined based on questionnaire responses. Participants who were diagnosed with diabetes by a healthcare professional or were currently undergoing diabetes treatment, including medication or insulin use, were classified as having a history of diabetes. Participants who were informed by a healthcare professional that they had hypertension or were currently receiving antihypertensive treatment were classified as having a history of hypertension. Cancer history was recorded for those who were diagnosed with cancer by a healthcare professional. Cardiovascular disease history included congenital heart disease, congestive heart failure, coronary artery disease, angina, or myocardial infarction, as diagnosed by a healthcare professional. Stroke history was recorded for participants who were diagnosed with a stroke by a healthcare professional. The systemic inflammatory markers included in this study were (1) CRP; (2) NLR =  Neutrophils/Lymphocytes; (3) PLR =  Platelets/Lymphocytes; (4) MLR =  Monocytes/Lymphocytes; (5) PWR =  Platelets/White Blood Cells; (6) SII = (Platelets × Neutrophils)/Lymphocytes; and (7) SIRI = (Neutrophils × Monocytes)/Lymphocytes.

### 2.4. Data Analysis

The baseline characteristics of the study population were described using weighted means ± standard errors (SEs), unweighted case numbers (*n*), and weighted percentages. The sample weights in NHANES data are designed to account for the complex survey design, ensuring that estimates are representative of the general US population. Multiple imputation was performed for missing covariates using chained equations, and all analyses were conducted in the imputed datasets incorporating survey weights. In this study, the Mobile Examination Center (MEC) data constituted the smallest subgroup containing all variables intended for analysis. Therefore, in accordance with NHANES guidelines, we used the 6‐year sample weights (MEC6YR) provided by the NCHS for the analysis. When combining data from the 2007–2008, 2009–2010, and 2011–2012 survey cycles, multi‐cycle sample weights were calculated by dividing the 2‐year weights by the number of 2‐year cycles included in the analysis. Specifically, the formula was as follows: if the survey cycle code was 5, 6, or 7, then MEC6YR = (1/3) × WTMEC2YR. Further details on weight creation can be found in the estimation procedure documents on the NHANES official website.

Study participants were divided into three groups based on tertiles of baseline FT3 levels. For continuous variables, weighted linear regression models were used for comparisons, while categorical variables were compared using weighted chi‐square tests. To evaluate the monotonic associations between FT3 and seven inflammatory markers (CRP, NLR, MLR, PLR, PWR, SII, and SIRI), Spearman rank correlation coefficients were calculated. Multiple weighted multiple linear regression models were constructed: Model 1 was adjusted for age, sex, and race; Model 2 was further adjusted for BMI, diabetes, heart disease, hypertension, stroke, cancer, smoking history, and alcohol consumption. In all models, FT3 tertiles were included as a continuous variable to test for trends. Additionally, stratified analyses were conducted by age (<65 years vs. ≥65 years), sex (male vs. female), race (non‐Hispanic White, non‐Hispanic Black, Mexican American or other Hispanic, and other), BMI (<25 kg/m^2^ vs. ≥25 kg/m^2^), TSH (divided into low and high groups based on median level), and FT4 (divided into low and high groups based on median level). The associations between FT3 and inflammatory markers were compared across these subgroups, and interaction tests were performed. The results are presented in forest plots. All statistical analyses were performed using R software (version 4.3.1) and EmpowerStats (https://www.empowerstats.com), with the significance level set at *α* = 0.05.

## 3. Results

### 3.1. Baseline Characteristics of the Study Population

A total of 2306 participants were included in this study and were divided into three groups based on tertiles of baseline FT3 levels (T1‐T3). The baseline characteristics of the participants were summarized in Table 1. With increasing FT3 levels, the proportion of males, DBP, FT4, ALT, triglycerides, uric acid, and white blood cell count progressively increased, with statistically significant differences between groups (*p* < 0.05). Age, SBP, TSH, HDL, fasting blood glucose, creatinine, BUN, and the proportion of females gradually decreased from T1 to T3 groups, with statistically significant intergroup differences (*p* < 0.05). Additionally, BMI, monocyte count, PLR, PWR, ethnicity, low‐density lipoprotein (LDL), and history of diabetes, heart disease, hypertension, and cancer showed statistically significant differences among groups (*p* < 0.05). In contrast, no statistically significant differences were observed between groups for AST, total cholesterol, uric acid, platelet count, neutrophil count, lymphocyte count, NLR, MLR, PWR, SII, SIRI, CRP, smoking history, alcohol consumption history, or history of stroke (*p* > 0.05).

**Table 1:** Baseline Characteristics of patients.

Characteristics	FT3 (pg/mL)			*p* Value
	T1 (1.9‐2.97) *n*=769	T2 (2.98‐3.29) *n*=768	T3 (3.3‐6.3) *n*=769	
Age (years)	56.80 (16.88)	49.56 (16.54)	43.13 (15.09)	**<0.001**
Gender (%)				
Male	299 (35.1)	334 (44.8)	466 (60.6)	**<0.001**
Female	470 (64.9)	434 (55.2)	303 (39.4)	
Race (*n*,%)				
Mexican American	373 (70.4)	355 (69.9)	283 (60.1)	**<0.001**
Non‐Hispanic White	187 (12.8)	155 (11.3)	125 (9.9)	
Non‐Hispanic Black	166 (9.1)	210 (12.4)	304 (21.3)	
Other Race	43 (7.7)	48 (6.5)	57 (8.7)	
BMI (kg/m^2^)	27.45 (6.14)	28.39 (7.10)	28.23 (6.01)	**0.05**
SBP (mmHg)	125.90 (20.36)	123.94 (17.37)	120.92 (15.72)	**<0.001**
DBP (mmHg)	68.97 (11.93)	71.00 (11.73)	71.99 (10.93)	**<0.001**
FT4 (pmol/L)	10.13 (1.75)	10.16 (1.65)	10.57 (1.82)	**<0.001**
TSH (uIU/mL)	1.95 (1.62)	1.83 (1.23)	1.73 (1.06)	**<0.001**
ALT (U/L)	21.23 (14.07)	22.62 (11.05)	27.17 (15.48)	**<0.001**
AST (U/L)	24.14 (8.36)	24.54 (8.12)	26.48 (17.44)	0.16
Cholesterol (mmol/L)	5.03 (1.05)	5.06 (1.08)	5.05 (1.05)	0.83
TG (mmol/L)	1.62 (1.25)	1.74 (1.40)	1.91 (1.59)	**0.03**
LDL (*n*,%)				
Abnormal (>3.1mmol/L)	83 (10.3)	128 (15.5)	128 (17.0)	**<0.001**
Normal (≤3.1mmol/L)	224 (29.4)	242 (29.7)	275 (34.8)	
Missing	462 (60.3)	398 (54.7)	366 (48.2)	
HDL (mmol/L)	1.45 (0.41)	1.35 (0.38)	1.24 (0.37)	**<0.001**
FBG (mmol/L)	5.67 (2.08)	5.63 (2.38)	5.40 (1.48)	**0.05**
Uric acid (umol/L)	311.20 (89.73)	314.73 (80.31)	329.85 (72.97)	**<0.001**
Creatinine (umol/L)	80.76 (43.90)	77.20 (25.42)	75.40 (16.91)	**<0.001**
BUN (mmol/L)	5.35 (2.41)	4.76 (1.87)	4.37 (1.41)	**<0.001**
CRP (*n*,%)				0.10
Abnormal (≥5mg/dL)	10 (0.9)	0 (0.0)	1 (0.0)	
Normal(<5mg/dL)	613 (70.6)	606 (69.8)	639 (76.2)	
Missing	146 (28.4)	162 (30.2)	129 (23.8)	
Platelet (10^12^/L)	254.41 (73.37)	249.76 (63.98)	252.60 (66.68)	0.63
WBC (10^9^/L)	6.82 (2.15)	7.10 (1.98)	7.15 (2.02)	**0.05**
Neutrophil (10^9^/L)	4.05 (1.55)	4.20 (1.60)	4.25 (1.66)	0.16
Monocyte (10^9^/L)	0.51 (0.19)	0.54 (0.18)	0.53 (0.15)	**0.05**
Lymphocyte (10^9^/L)	2.03 (1.07)	2.11 (0.67)	2.12 (0.65)	0.07
NLR	2.27 (1.31)	2.15 (1.01)	2.14 (0.99)	0.10
MLR	0.28 (0.14)	0.28 (0.12)	0.27 (0.10)	0.16
PLR	142.80 (65.52)	127.55 (45.68)	127.88 (45.62)	**0.01**
PWR	39.56 (13.91)	36.95 (11.12)	37.38 (12.22)	**0.05**
SII	576.92 (367.72)	535.35 (277.64)	542.80 (309.55)	0.18
SIRI	0.16 (0.14)	0.16 (0.12)	0.15 (0.09)	0.40
Pre‐existing medical conditions				
Diabetes (*n*,%)				
Yes	165 (15.5)	116 (13.2)	86 (7.1)	**<0.001**
No	605 (84.4)	652 (86.8)	682 (92.8)	
Missing	2 (0.2)	0 (0.0)	1 (0.1)	
Heart disease (*n*,%)				
Yes	112 (11.1)	63 (6.5)	33 (3.8)	**<0.001**
No	653 (88.6)	703 (93.4)	733 (96.0)	
Missing	4 (0.3)	2 (0.1)	3 (0.2)	
Hypertension (*n*,%)				
Yes	299 (31.9)	224 (22.7)	162 (16.3)	**<0.001**
No	307 (43.3)	358 (48.1)	431 (54.5)	
Missing	163 (24.8)	186 (29.1)	176 (29.2)	
Stroke (*n*,%)				
Yes	53 (5.0)	37 (3.3)	23 (2.2)	0.12
No	715 (94.9)	731 (96.7)	746 (97.8)	
Missing	1 (0.1)	0 (0.0)	0 (0.0)	
Cancer (*n*,%)				
Yes	123 (15.3)	79 (10.0)	45 (5.6)	**<0.001**
No	645 (84.4)	689 (90.0)	722 (94.3)	
Missing	1 (0.2)	0 (0.0)	2 (0.1)	
Smoking status (*n*,%)				
Yes	335 (40.1)	300 (41.3)	319 (41.1)	0.85
No	434 (59.9)	467 (58.7)	449 (58.8)	
Missing	0 (0.0)	1 (0.0)	1 (0.1)	
Alcohol use (*n*,%)				
Yes	128 (15.0)	111 (12.1)	118 (14.6)	0.38
No	128 (12.3)	153 (15.8)	135 (14.9)	
Missing	513 (72.7)	504 (72.1)	516 (70.5)	

*Note*: Bold value signifies the statistically significant variables clearly distinguishable from those that are not.

Abbreviations: ALT, alanine aminotransferase; AST, aspartate aminotransferase; BMI, body mass index; BUN, blood urea nitrogen; CRP, C‐reactive protein; DBP, diastolic blood pressure; FBG, fasting blood glucose; FT3, free triiodothyronine; FT4, free thyroxine; HDL, high‐density lipoprotein; LDL, low‐density lipoprotein cholesterol; MLR, monocyte‐to‐lymphocyte ratio; NLR, neutrophil‐to‐lymphocyte ratio; PLR, platelet‐to‐lymphocyte ratio; PWR, platelet‐to–white blood cell ratio; SBP, systolic blood pressure; SII, systemic immune–inflammation index; SIRI, systemic inflammatory response index; TG, triglycerides; TSH, thyroid‐stimulating hormone; WBC, white blood cell count.

### 3.2. Univariate Correlation Analysis of the Association Between FT3 and Inflammatory Markers

Figure 2 demonstrates the monotonic associations between various inflammatory markers and FT3 levels. NLR, MLR, and PLR all exhibited a significant negative correlation with FT3 levels, which was statistically significant (*p* < 0.05). PWR, SIRI, SII, and CRP showed nonsignificant negative associations (all *p*  > 0.05).

Figure 2Correlation analyses of free triiodothyronine (FT3) with various inflammatory markers. (a) Neutrophil‐to‐lymphocyte ratio (NLR), (b) monocyte‐to‐ lymphocyte ratio (MLR), (c) platelet‐to‐lymphocyte ratio (PLR), (d) platelet‐to‐white blood cell ratio (PWR), (e) systemic inflammation response index (SIRI), (f) systemic immune‐inflammation index (SII), and (g) C‐reactive protein (CRP). The scatter points represent individual observations, the blue solid line indicates the trend line fitted based on linear regression, and the gray shaded area represents the 95% confidence interval. The Spearman correlation coefficient (R) and *p*‐value displayed above each subplot reflect the strength and statistical significance of the linear association between FT3 and the respective inflammatory marker. All analyses were weighted using NHANES survey weights to ensure national representativeness (*n* = 2306).

**Figure:**
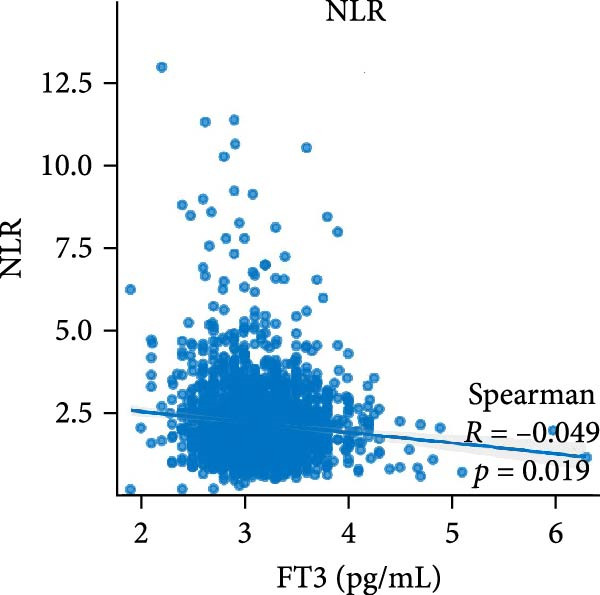
(a)

**Figure:**
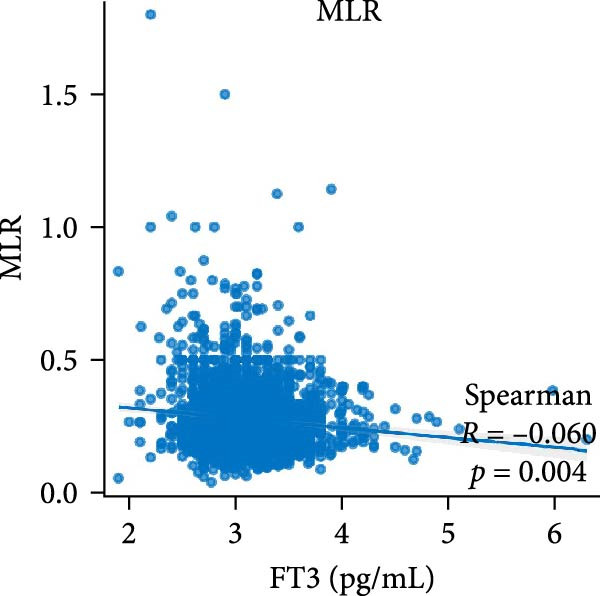
(b)

**Figure:**
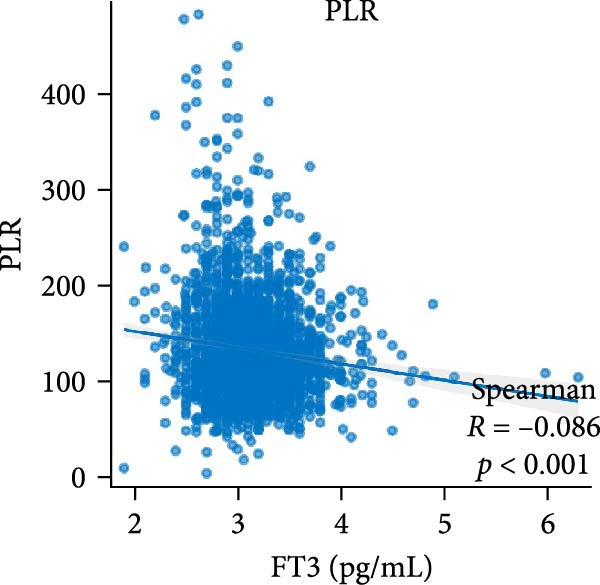
(c)

**Figure:**
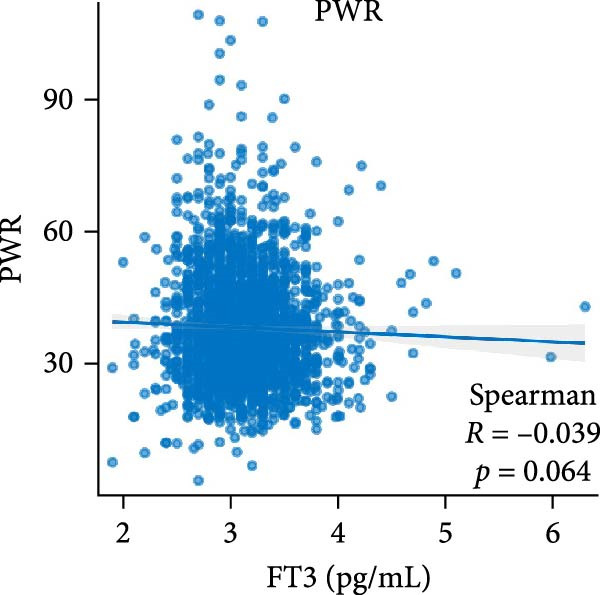
(d)

**Figure:**
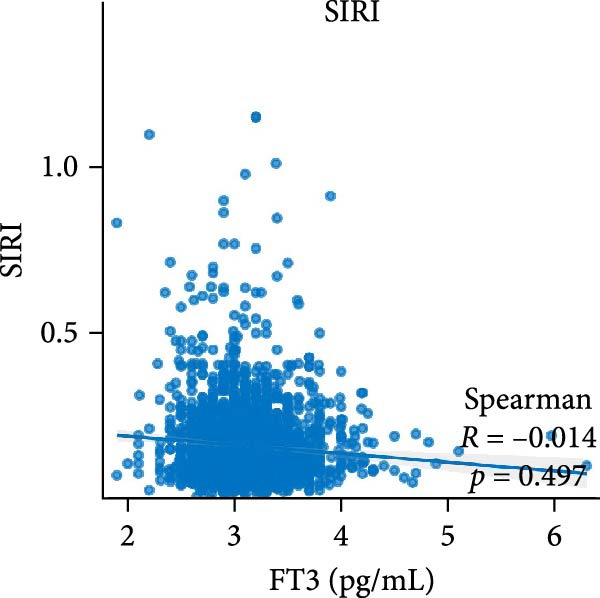
(e)

**Figure:**
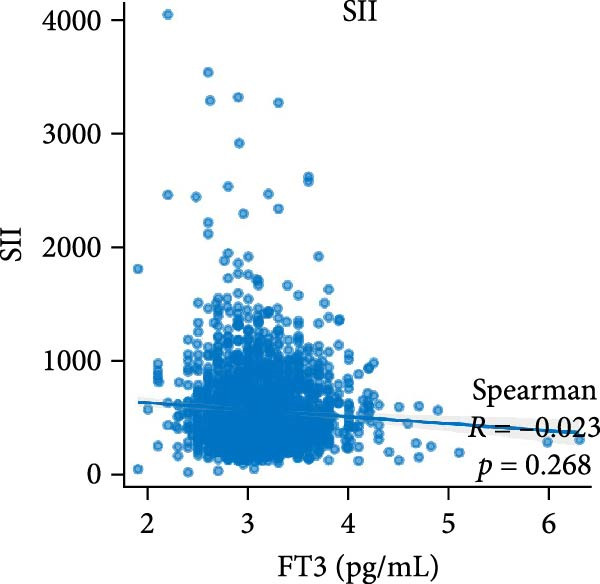
(f)

**Figure:**
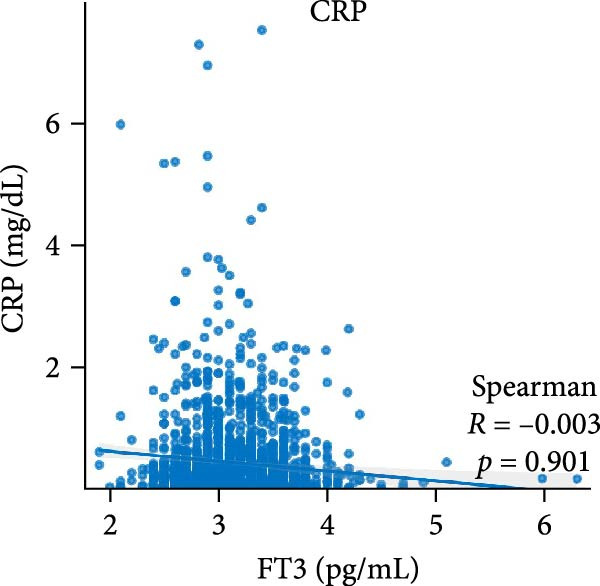
(g)

### 3.3. Multivariate Regression Analysis of the Association Between FT3 and Inflammatory Markers

According to the STROBE statement, this study sequentially constructed unadjusted models, Model 1, and Model 2 to explore the stable association between FT3 and inflammatory markers. In the unadjusted model, FT3 exhibited a negative association with all inflammatory markers, which remained significant even when FT3 was categorized into tertiles, with trend tests showing statistical significance (all *p*  < 0.05). In the minimally adjusted Model 1, FT3 maintained a significant negative association with NLR and PWR (all *p*  < 0.05). In Model 2, after adjusting for all potential confounders, FT3 remained significantly associated with CRP, NLR, and PLR (all *p*  < 0.05). Overall, FT3 exhibited a consistent negative association with inflammatory markers (Table 2).

**Table 2:** Linear regression of FT3 with inflammatory markers.

Variable	Crude model	Model 1	Model 2
	*β* (95%CI *p* Value	*β* (95%CI *p* Value	*β* (95%CI *p* Value
CRP			
FT3 (pg/mL)	−0.08 (−0.20, −0.04) **0.048**	−0.03 (−0.11, 0.17) 0.648	−0.01(−0.03, 0.00) 0.145
FT3 group			
Low	0	0	0
Middle	−0.11 (−0.19, −0.02) 0.017	−0.07 (−0.16, 0.01) 0.078	−0.04 (−0.12, 0.05) 0.374
High	−0.14 (−0.18, −0.04) <0.001	−0.01 (−0.09, 0.08) 0.887	−0.10 (−0.20, −0.01) 0.037
*p* for trend	**<0.001**	0.919	**0.028**
NLR			
FT3 (pg/mL)	−0.20 (−0.35, −0.05) **0.012**	−0.09 (−0.26, 0.07) 0.261	−0.11 (−0.21, −0.01) **0.038**
FT3 group			
Low	0	0	0
Middle	−0.13 (−0.26, 0.01) 0.064	−0.14 (−0.22, −0.06) < 0.001	−0.08 (−0.14, −0.02) 0.010
High	−0.13 (−0.27, −0.07) 0.012	−0.17(−0.22, −0.11) <0.001	−0.07 (−0.13, −0.01) 0.025
* p* for trend	**0.002**	**<0.001**	**0.001**
MLR			
FT3 (pg/mL)	−0.03 (−0.05, −0.01) **0.006**	−0.00 (−0.03, 0.02) 0.684	−0.00 (−0.02, 0.02) 0.82
FT3 group			
Low	0	0	0
Middle	−0.01 (−0.02, 0.01) 0.318	0.00 (−0.01, 0.02) 0.789	0.00 (−0.01, 0.02) 0.618
High	−0.02 (−0.03, −0.01) <0.001	0.00 (−0.02, 0.02) 0.826	0.00 (−0.01, 0.02) 0.643
* p* for trend	**<0.001**	0.828	0.649
PLR			
FT3 (pg/mL)	−12.86 (−13.37, −12.35) **<0.001**	0.73 (−13.12, 14.57) 0.916	−9.80 (−18.96, −0.63) **0.037**
FT3 group			
Low	0	0	0
Middle	−11.8 (−16.49, −7.18) <0.001	−3.71 (−14.88, 7.45) 0.506	−11.22 (−21.61, −0.83) 0.03
High	−12.5 (−14.31, −10.68) <0.001	0.21 (−12.77, 13.18) 0.975	−8.45 (−18.19, −0.37) 0.041
* p* for trend	**<0.001**	0.966	**0.011**
PWR			
FT3 (pg/mL)	−1.91 (−3.77, −0.04) **0.045**	−1.16 (−1.90, −0.43) **0.002**	−1.03 (−3.13, 1.07) 0.323
FT3 group			
Low	0	0	0
Middle	−2.61 (−4.85, −0.37) 0.023	−2.25 (−4.43, −0.07) 0.043	−2.04 (−4.35, 0.27) 0.082
High	−2.18 (−4.03, −0.33) 0.022	−1.42 (−2.75, −0.10) 0.036	−1.22 (−3.34, 0.89) 0.246
* p* for trend	**0.024**	**0.013**	0.255
SII			
FT3 (pg/mL)	−48.40 (−102.24, 5.44) 0.077	−23.21 (−80.75, 34.33) 0.42	−31.80 (−90.94, 27.34) 0.28
Low	0	0	0
Middle	−44.06 (−85.62, −2.47) 0.041	−28.81 (−73.05, 15.42) 0.196	−33.30 (−79.07, 12.46) 0.147
High	−52.30 (−86.41, −18.18) 0.003	−11.44 (−68.66, 45.77) 0.689	−18.27 (−77.11, 40.58) 0.53
* P* for trend	**<0.001**	0.699	0.543
SIRI			
FT3 (pg/mL)	−0.02 (−0.04, −0.00) **0.038**	0.00 (−0.02, 0.02) 0.991	0.00 (−0.02, 0.02) 0.838
FT3 group			
Low	0	0	0
Middle	−0.01 (−0.02, 0.00) 0.049	0.01 (−0.00, 0.03) 0.163	0.01 (−0.00, 0.03) 0.13
High	−0.01 (−0.02, 0.00) 0.047	0.01 (−0.01, 0.02) 0.375	0.01 (−0.00, 0.03) 0.274
*p* for trend	**<0.001**	0.391	0.29

*Note:* Bold value signifies the statistically significant variables clearly distinguishable from those that are not. Model 1 was adjusted for age, sex, and race. Model 2 was adjusted for age, sex, race, BMI, diabetes, heart disease, hypertension, stroke, cancer, and history of smoking and alcohol use.

Abbreviations: CRP, C‐reactive protein; FT3, free triiodothyronine; MLR, monocyte‐to‐lymphocyte ratio; NLR, neutrophil‐to‐lymphocyte ratio; PLR, platelet‐to‐lymphocyte ratio; PWR, platelet‐to‐white blood cell ratio; SII, systemic immune‐inflammation index; SIR, systemic inflammatory response index.

### 3.4. Subgroup Analysis of the Association Between FT3 and Inflammatory Markers

Figure 3 presents forest plots depicting the associations of FT3 with seven inflammatory markers (CRP, NLR, MLR, PLR, PWR, SII, and SIRI), stratified by age (< 65 and ≥ 65 years), sex, race/ethnicity (non‐Hispanic White, non‐Hispanic Black, Mexican American or other Hispanic, and other), BMI (< 25 and ≥ 25 kg/m^2^), TSH (categorized by median), and FT4 (categorized by median), along with tests for interaction. Significant interaction effects were observed for the association between FT3 and CRP by both age and sex (*p*‐interaction < 0.05) and for the associations between FT3 and NLR, MLR, SII, and SIRI by age (all *p*‐interaction < 0.05). In contrast, no significant interactions were found for the associations of FT3 with PWR or PLR across any of the subgroups examined (*p*‐interaction ≥ 0.05). Notably, among older participants (≥ 65 years), the inverse correlations of FT3 with CRP, NLR, MLR, SII, and SIRI were more pronounced.

Figure 3Association of FT3 levels with inflammatory markers across demographic and clinical subgroups. (a) C‐reactive protein (CRP), (b) neutrophil‐to‐lymphocyte ratio (NLR), (c) monocyte‐to‐ lymphocyte ratio (MLR), (d) platelet‐to‐lymphocyte ratio (PLR), (e) platelet‐to‐white blood cell ratio (PWR), (f) systemic immune‐inflammation index (SII), and (g) inflammation response index (SIRI). Results are presented as β coefficients with 95% confidence intervals (95% CI), and the dashed vertical line in the figure indicates the null effect line (*β* = 0). These results were derived from weighted multivariable linear regression models. Interaction *p*‐values were used to test for effect modification across different subgroups. All models have been adjusted for age, gender, race, BMI, diabetes, heart disease, hypertension, stroke, cancer, smoking, and drinking history. Stratified analyses were performed without further adjustment for the stratification variables.

**Figure:**
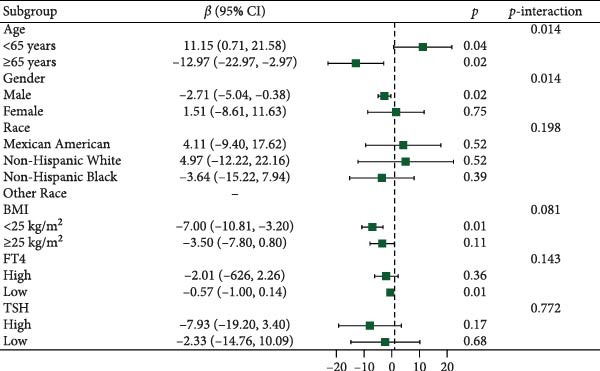
(a)

**Figure:**
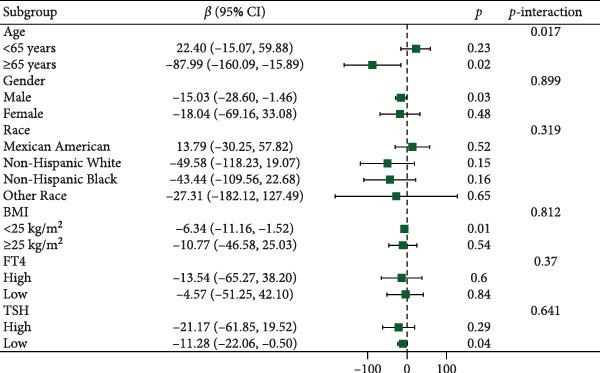
(b)

**Figure:**
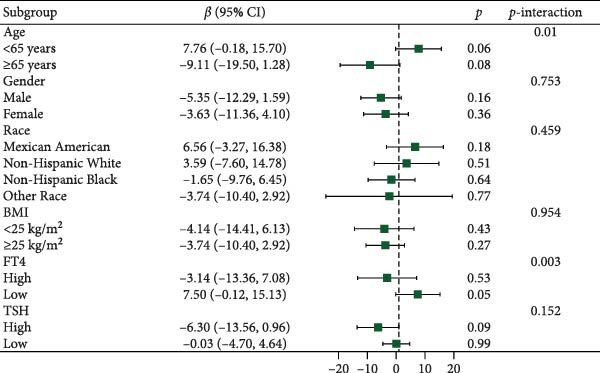
(c)

**Figure:**
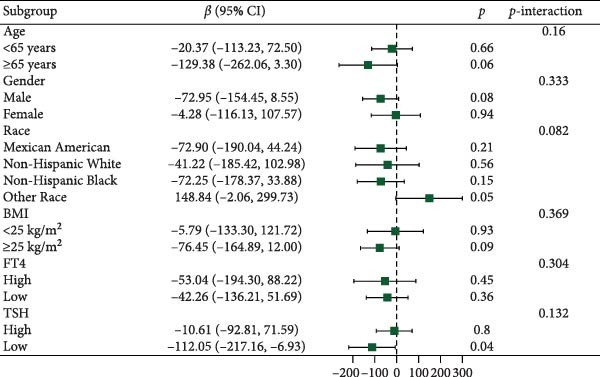
(d)

**Figure:**
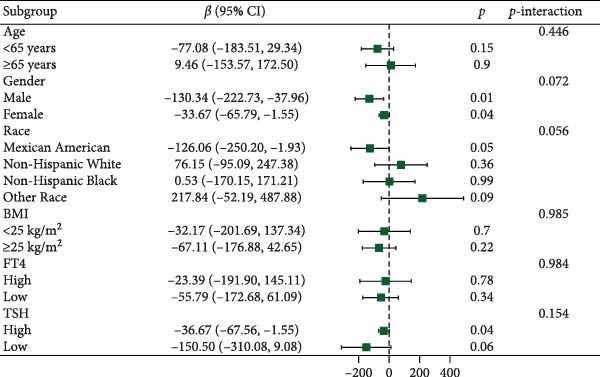
(e)

**Figure:**
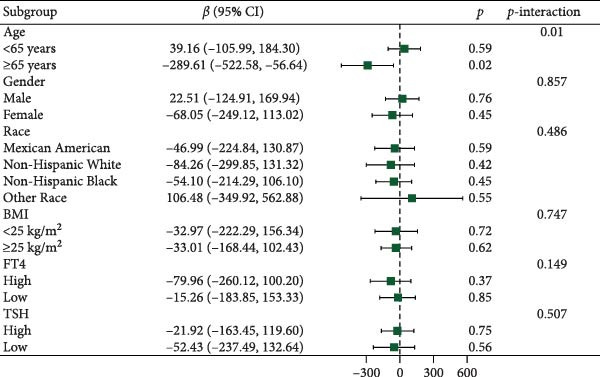
(f)

**Figure:**
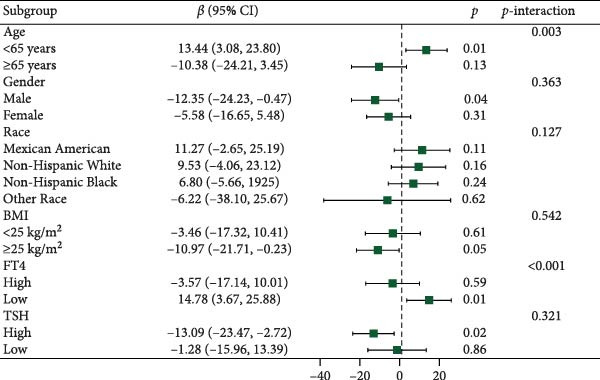
(g)

### 3.5. Supplementary Materials

Before modeling, collinearity diagnostics were performed on the covariates, and no severe multicollinearity was detected (all VIF < 5, Supporting Information Table S1). Table S1 lists the VIF for all baseline variables, including FT3, age, gender, race, BMI, and clinical history indicators (diabetes, heart disease, hypertension, stroke, cancer, smoking, and alcohol use).

## 4. Discussion

This study systematically examined the association between FT3 and multiple systemic inflammatory markers using data from 2306 adults in NHANES 2007–2012. We found significant inverse associations between FT3 and CRP, NLR, LMR, PLR, PWR, SII, and SIRI, which remained robust for CRP, NLR, and PLR after adjusting for potential confounders—including age, sex, race, BMI, diabetes, cardiovascular disease, hypertension, stroke, cancer, smoking, and alcohol use. Our population‐based findings extend prior observations from clinical cohorts. For example, studies in patients with type 2 diabetes reported inverse associations between FT3 and NLR or PLR only [26], while, in acute myocardial infarction, lower FT3 levels were linked to elevated CRP and poorer outcomes, partially mediated by inflammation [24]. Compared with previous work, this study has several strengths. First, by including individuals regardless of disease status, our design minimizes bias from advanced comorbidities or disease‐specific treatments. Second, to our knowledge, this is the first study to simultaneously assess FT3 in relation to seven distinct inflammatory markers in a nationally representative sample, offering broader biomarker coverage and improved generalizability.

The selection of these seven inflammatory markers was guided by their routine availability in NHANES, where CBC and CRP are regularly measured, as well as by their clinical accessibility and low cost, thereby enhancing the translational relevance of our findings. Although nonspecific and not reflective of localized immune activation [29], these indices collectively capture key aspects of systemic inflammation and immune‐coagulation crosstalk. NLR is a well‐validated indicator of infection and inflammation [30, 31]; LMR and SIRI reflect chronic immune activation; PLR and PWR incorporate platelet reactivity, linking coagulation and inflammation. CRP, as a hepatic acute‐phase protein, provides a general measure of inflammatory burden [32–34]. Together, this multiparameter approach mitigates the limitations of single biomarkers and enables a more comprehensive assessment of immune‐inflammatory pathways. The observed heterogeneity in FT3 associations across markers suggests that thyroid function may modulate distinct inflammatory mechanisms through diverse biological pathways. Future longitudinal or interventional studies with dynamic hormone and inflammation monitoring are needed to clarify causal relationships and clinical implications.

In the subgroup analysis, our study found that age significantly influenced the associations between FT3 and several inflammatory markers, such as CRP, NLR, MLR, SII, and SIRI, with these inverse relationships being more evident among older adults aged 65 years and above. This observation can be interpreted in two ways. First, aging is commonly associated with a persistent, low‐grade activation of the immune system, referred to as inflammaging [35, 36]. Second, FT3 levels naturally tend to decrease with age [37]. As a result, the pattern of low FT3 coupled with high inflammation becomes more prominent in the elderly population. These findings carry potential clinical significance. The stronger associations observed in older adults suggest that FT3 could be used as an additional biomarker to evaluate chronic inflammation and overall health risk in this age group. Although this study did not directly assess mortality outcomes, a large body of evidence has consistently linked low FT3 levels to higher risks of all‐cause and cardiovascular mortality [38, 39]. Therefore, integrated monitoring of thyroid function and systemic inflammation in older individuals, particularly those with preexisting metabolic or cardiovascular diseases, may aid in the early identification of high‐risk patients.

The mechanisms underlying endocrine–immune interactions remain incompletely understood. Current evidence indicates that proinflammatory cytokines (e.g., TNF‐α, IL‐1, IL‐6, and IFN‐γ) suppress TH levels by inhibiting the hypothalamic‐pituitary‐thyroid axis and disrupting deiodinase activity, thereby reducing FT3 [10, 40]. Conversely, T3 exerts anti‐inflammatory effects by binding nuclear receptors and modulating key signaling pathways, including NF‐κB, p38/MAPK, and JAK/STAT [41]. It also regulates immune cell function, particularly that of macrophages, through metabolic reprogramming and epigenetic modifications, thereby influencing the production of inflammatory mediators and fine‐tuning systemic inflammation [42]. These bidirectional interactions provide a plausible biological basis for the inverse association between FT3 and systemic inflammatory markers observed in our study, highlighting the intricate crosstalk between the endocrine and immune systems at the systemic level.

Certainly, this study has several limitations. First, the cross‐sectional design limits causal inference, as it remains unclear whether low FT3 leads to elevated inflammation, chronic inflammation suppresses thyroid function, or both are driven by shared unmeasured confounding factors. Second, although we adjusted for a range of known confounders, residual biases from unmeasured variables may still exist, such as medication use (e.g., corticosteroids, anti‐inflammatory drugs), subclinical infections, and nutritional status, all of which could influence the observed results. Furthermore, due to the strong physiological interdependence among FT3, TSH, and FT4, the potential confounding influence of TSH and FT4 cannot be completely excluded, despite our efforts to assess potential effect modification via subgroup analyses. Third, the NHANES database does not systematically include more specific inflammatory mediators such as ESR and IL‐6. As a result, these key markers were not included in the analysis, which restricts further exploration of potential molecular mechanisms. Fourth, the sample size is relatively limited; some inflammatory markers lost statistical significance after full multivariable adjustment, which may indicate insufficient statistical power. Future studies should be conducted in larger prospective cohorts and should incorporate high‐sensitivity inflammatory profiles along with clinical outcomes such as mortality and cardiovascular events to further clarify the role of FT3 in inflammatory regulation.

## 5. Conclusion

This study demonstrates an inverse correlation between FT3 levels and systemic inflammatory markers in the general population, suggesting that FT3 may serve as a potential biomarker for inflammation. These findings provide novel insights for the prevention and management of inflammatory diseases, while facilitating improved risk stratification. Furthermore, our results highlight the clinical importance of enhanced monitoring and preventive measures for patients with hypothyroidism comorbid with chronic inflammatory conditions, including autoimmune diseases, infections, cardiovascular disorders, obesity, and diabetes mellitus.

## Conflicts of Interest

The authors declare no conflicts of interest.

## Author Contributions

All authors contributed to the study conception and design. Data collection and analysis: Liu Yang, Han‐yu Wang, and Meng‐fei Fu. Software and visualization: Yu‐han Zhang, Xiao Chen, and Zi‐xuan Wang. Writing – original draft preparation: Liu Yang. Writing – review and editing: all authors. Supervision: Hui Sun. Liu Yang and Han‐yu Wang contributed equally to this work and share first authorship.

## Funding

This work was supported by the National Natural Science Foundation of China (Grant Number 82270832).

## Supporting Information

Additional supporting information can be found online in the Supporting Information section.

## Supporting information


**Supporting Information** Supporting Information Table 1: Variance Inflation Factor (VIF) values for baseline characteristics, including free triiodothyronine (FT3), age, gender, race, body mass index (BMI), and history of diabetes, heart disease, hypertension, stroke, cancer, smoking, and alcohol use. All VIF values were < 5, indicating no substantial multicollinearity among predictors.

## Data Availability

The data that support the findings of this study are available from the corresponding author upon reasonable request.
