# Supplementary material for: FT3 Levels and Systemic Inflammation: Evidence From a Population‐Based NHANES Analysis
Source: Mediators Inflamm. 2026 Jan 26;2026:3764432. doi: 10.1155/mi/3764432 (PMC12835195; doi:10.1155/mi/3764432)
Supplement: Supplementary file 1 — Supporting Information Supporting Information Table 1: Variance Inflation Factor (VIF) values for baseline characteristics, including free triiodothyronine (FT3), age, gender, race, body mass index (BMI), and history of diabetes, heart disease, hypertension, stroke, cancer, smoking, and alcohol use. All VIF values were < 5, indicating no substantial multicollinearity among predictors. [file MI-2026-3764432-s001.docx]

**Supplementary Table 1** Baseline Indicator Collinearity Test

|  | VIF |
| --- | --- |
| FT3 | 1.2 |
| Age | 1.6 |
| Gender | 1.1 |
| Race | 1.1 |
| BMI | 1.1 |
| Diabetes | 1.2 |
| Heart disease | 1.1 |
| Hypertension | 1.2 |
| Stroke | 1.1 |
| Cancer | 1.1 |
| Smoking | 1.1 |
| Alcohol use | 1.1 |

**Abbreviations**: FT3: Free Triiodothyronine; VIF: Variance Inflation Factor; BMI: Body Mass Index
